# Delivering Better Oral Health: keep building evidence

**DOI:** 10.1038/s41415-026-9884-3

**Published:** 2026-11-13

**Authors:** Jennifer E. Gallagher, Ivor G. Chestnutt, Jan Clarkson, David I. Conway, Anne-Marie Glenny, Rebecca V. Harris, Zoe Marshman, Laura McDonald, Peter G. Robinson, Douglas Stirling, Jenny Godson

**Affiliations:** 41415808583001 King´s College London, Faculty of Dentistry, Oral and Craniofacial Surgery, Centre for Host Microbiome Interactions (Dental Public Health), London, UK; 41415808583002 Cardiff University, Dental Public Health, Cardiff, UK; 41415808583003 University of Dundee, Dundee, UK; 41415808583004 University of Glasgow, School of Medicine, Dentistry and Nursing, Glasgow, UK; 41415808583005 University of Manchester, Division of Dentistry, School of Medical Sciences, Manchester, UK; 41415808583006 University of Liverpool, Health Services Research, Liverpool, UK; 41415808583007 School of Clinical Dentistry, University of Sheffield, Oral Health and Development, Sheffield, UK; 41415808583008 Cochrane Oral Health, Division of Dentistry, The University of Manchester, Manchester, UK; 41415808583009 Dentistry, Bristol, UK; 41415808583010 Formerly NHS Education for Scotland, Edinburgh, UK; 41415808583011 Formerly Public Health England, Bradford, UK

## Abstract

Guidelines are systematically developed statements of best practice, informed by available evidence. Clinical practice guidelines play a particularly important role in contemporary healthcare facilitating busy clinicians to make sense of the large, diverse, and rapidly growing body of research evidence. Delivering Better Oral Health is now in its fourth version and available as an online digital resource. To ensure that we continually build our evidence base on prevention, areas for further research relating to dental caries, oral cancer, periodontal disease, tooth wear and behaviour change were identified and compiled retrospectively to inform future research. National research funding should, ideally, support addressing these priority areas as part of an integrated plan.

## Key points

- Delivering Better Oral Health provides evidence-based guidance for all members of the dental team to support the care and management of all patients, based on disease risk.
- While there is a strong body of evidence on the prevention of oral disease, this paper provides insights to helpful areas for further research.
- Researchers and clinicians are encouraged to further build the evidence in support of promoting oral health.

## Introduction

Guidelines are systematically developed statements of best practice, informed by available evidence. Clinical practice guidelines play a particularly important role in contemporary healthcare facilitating busy clinicians to make sense of the large, diverse, and rapidly growing body of research evidence.

Delivering Better Oral Health: an evidence-based guide to prevention, first published in 2007,^1^ is now in its fourth edition (DBOHv4).^2^ Each successive version,^3^^,^^4^^,^^5^ has drawn on systematic reviews, newly published research, and other relevant guidelines to provide front line providers with a compilation of the latest contemporary evidence to inform patient care.

The development of Version 4, which will be referred to as DBOHv4 in this article, represented a significant change in approach using contemporary methodology within a structured process.^6^ It drew on the wide-ranging views of stakeholders and experts across the UK as contributors in a three-tier governance structure.^6^ First, oversight was provided by professional leaders who provided high-level strategic guidance. Second, the middle tier Guideline Working Group was the powerhouse for the project. Third, five short-term ‘Guideline Development Groups' addressed dental caries, periodontal diseases, tooth wear, oral cancer and behaviour change in turn, considering risk and protective factors. The final document is a resource for all dental professionals in providing contemporary evidence-informed care.

## Evidence base

During the development of DBOHv4, the relevant body of research evidence was reviewed in a rigorous manner to address several key research questions that had been approved by each Guideline Development Group,^6^ in relation to dental caries, periodontitis, oral cancer, tooth wear and behaviour change. Evidence searches were devised that focused on finding systematic reviews and clinical guidelines to answer key questions related to the specific disease/condition and its risk management. The resultant key guidance statements are presented in the summary tables of Chapter 2, of DBOHv4, with more detailed information in relation to the strength of evidence available to readers in Chapter 13. New emerging issues, on which there was less evidence, are addressed in the text of successive chapters. The level of certainty we can have in the evidence was assessed according to GRADE,^6^^,^^7^ an internationally recognised tool for grading the quality of evidence and for making recommendations, in line with AGREE principles.^6^^,^^8^

## Building further evidence

The aim of this paper is to highlight priority areas where evidence on the clinical prevention of oral diseases could be further strengthened, or gaps filled, in support of future oral health guidelines. The material compiled and presented in this paper represents the views, expertise and experience of the authors who were all members of the Guideline Working Group. It was informed by their considerations of the available evidence, discussions within the series of workshops, and refined in a dedicated post-guidance review session. In doing so it is anticipated that this paper will encourage researchers, funders, and front-line service providers to design, fund, support and disseminate the necessary research. The key areas identified complement aspects of the work of the James Lind Alliance,^9^ which undertook an exercise to identify priority areas for the National Institute for Health and Care Research (NIHR) funding in the UK, and are presented for discussion and action, starting with dental caries the most prevalent condition across the life course.^10^ All questions are listed in Table 1.

**Table 1:** Research questions to build evidence on prevention

**Disease/condition**	**No**	**Evidence to be strengthened developed further, conditional evidence that could be built upon, e.g., research among different groups**
Dental caries	1	Which combination of fluoride delivery mechanisms work best in high-risk patients?
	2	Is there a role for sugar-free chewing gum?
	3	Is dried fruit suitable to recommend as a snack?
	4	How to best manage dietary sugar intake across the life course
Periodontitis	5	Does telling people to remove plaque make any difference? If so, how? (sub-questions below)
		a. Advise best methods of plaque removal to prevent gingivitis, achieve lowest risk of periodontitis and tooth loss
		b. Use behaviour change methods with oral hygiene instruction
		c. Assess patient's/parent/carer's preferences for plaque control; and
		d. Instruction on interdental cleaning.
	6	Does diabetic control improve periodontal health and conversely, do good personal and professional periodontal care improve diabetic outcomes?
	7	How effective is attending for regular checks of the health of gum and bone and professional oral hygiene around implants in the prevention and early detection of peri-implantitis?
Oral cancer	8	What is the effectiveness of smoking cessation (including the role of vaping) in dental settings?
	9	What is the most appropriate prevention bundle of care for patients following oral cancer treatment?
	10	How can referral pathways for patients with suspected oral cancer be improved?
Tooth wear	11	What are the dietary causes of dental erosion?
	12	What preventive interventions work for tooth wear *in vivo?*
	13	What index or indices are most appropriate for screening for and monitoring of tooth wear in a primary care setting
Behaviour change	14	What brief behaviour change interventions (for delivery by primary dental teams) can reduce consumption of sugar in different groups?
	15	How best to optimise the implementation of behaviour change interventions using primary dental teams and skill mix
	16	What intervention(s) can improve routine attendance by vulnerable (inclusion) groups within the population?
Other strategic issues	17	How can oral disease best be prevented in vulnerable groups especially older people as part of holistic care?
	18	General health/oral health links
	19	How do we best implement prevention guidance?
	20	How best to use skill mix within the dental team to deliver prevention

### Dental caries

During the deliberations of the dental caries guideline development group (GDG) there were several questions where a definitive answer would have been useful, but the evidence base was lacking. They relate to both fluoride and sugar, which are the preventive/risk factors for dental caries,^11^ as outlined below and presented in Table 1. While there is a great deal of research evidence on the role of fluoride in the clinical prevention of dental caries, there is a clear and urgent need to work on diet, notably sugar. Given the prevalence of dental caries, four challenging questions are outlined below.

#### 1. Which combination of fluoride delivery mechanisms work best in high-risk patients?

Evidence on the effectiveness of fluoride in the prevention of dental caries is incontrovertible.^12^^,^^13^^,^^14^^,^^15^ The effectiveness of various vehicles for the delivery of fluoride is also well understood, as is their relative effectiveness. However, when an individual is deemed at high caries-risk, there is no clarity over ‘which combination of fluoride delivery vehicles works best in high-risk patients' at different points in the life course?'.

It is accepted that everyone who has natural teeth benefits from twice daily toothbrushing with an age-appropriate standard fluoride toothpaste (up to 1,350–1,500 ppm fluoride). However, if on clinical examination and risk assessment, an individual is deemed in need of additional fluoride, whether this is best addressed using fluoride varnish, or fluoride mouthwash, or simply toothpaste with a higher than standard fluoride content is unclear. Furthermore, the potential for silver diamine fluoride to prevent as well as arrest disease is also important to address. This issue of the selection of fluoride therapy is likely to vary by patient choice, age, access to a dental professional, cost, and wider environmental factors; most notably, whether there is fluoride present in the public water supply. This is an area where the evidence base is rapidly expanding,^16^^,^^17^ and reliable evidence synthesis needed to inform guidance development. The timely inclusion of the latest evidence is a challenge for guideline development generally, including DBOH, and adapting methods to take account of this introduces the concept of living guidelines.

By an extrapolation of our understanding of the dose response of fluoride in preventing dental caries, fluoride toothpastes with 2,800 and 5,000 ppm fluoride concentration have been marketed as preventive agents for those deemed at higher caries risk. Many countries have only one formulation of high fluoride toothpaste, whereas the UK has both,^18^ and thus a clinical decision is required over which level of higher fluoride toothpaste is appropriate. DBOHv4, has clarified that the concept underlying the use of such toothpastes is not that they are used in perpetuity, but rather that they should normally be used for a limited period to help stabilise and reduce caries risk while dietary management is undertaken to reduce sugar intake, however how long this takes. Deepening our understanding about which patients are best suited for receiving high fluoride toothpaste, as opposed to an alternative fluoride regimen, requires further research. The (soon to be published) findings of the NIHR-funded REFLECT trial,^19^ evaluating the effectiveness and cost benefit of prescribing high dose fluoride toothpaste in preventing and treating caries, will provide evidence to consider at the next update.

#### 2. Is there a role for sugar-free chewing gum?

The use of sugar-free chewing gum has been advocated as a caries preventive agent for many years. It is suggested that chewing gum stimulates saliva flow, increases the buffering capacity of saliva, and helps dilute and remove sugars from the oral cavity. However, uncertainty remains as to the true place for the use of sugar-free gum in caries prevention. Limited research has been conducted on chewing gum as a vehicle for delivery of caries preventive/remineralising agents such as xylitol and fluoride, but the role of such products is similarly unclear. A significant consideration is whether it has social acceptability. Chewing gum meets with disapproval in some sections of society and the costs of cleaning up carelessly deposited gum,^20^ is a frequently cited social barrier to its use.^21^

#### 3. Is dried fruit suitable to recommend as a snack?

One question that has recurred in successive issues of DBOH relates to the cariogenic potential of dried fruit and whether it is safe to recommend it as a snack for children or, as advised in DBOHv4, only as part of a meal? Dried fruit is rich in sugars and is retentive in nature. Yet it is accepted as having some nutritional value as one of the 5-A-Day challenge,^22^ and thus it may be an acceptable component of a child's lunchbox. From a theoretical perspective it seems logical not to recommend eating dried fruit as a snack. While fresh fruit would seem like a preferred alternative as a between-meals snack, to minimise the frequency of eating sugars, the convenience and commercial marketing of dried fruit mean that those involved in health education will likely continue to face questions on the suitably of dried fruit as a snack that is safe for teeth, particularly as 30g is acceptable as one portion of fruit per day by nutritionists.^23^

#### 4. How to best manage dietary sugar intake across the life course

There are many important research areas in relation to sugar intake which is a risk factor for oral and general health problems. Across the life course, dietary public health interventions could helpfully consider oral health as well as general health in tackling obesity and other con-communicable diseases as part of an integrated risk management approach. This should start with consideration of how to best help parents to build healthy dietary patterns for their children from birth. There are other interesting influences, including the fact that eating in most societies is a communal activity. In all aspects of caries prevention research, consideration needs to be given to the context and section of society involved, as dietary preferences and patterns differ.^24^ This would be assisted by research into better self-reported dietary measures of sugar intake, and frequency, in support of oral health.

### Periodontal diseases

The most common forms of periodontal diseases, ‘gingivitis' (inflammation of the gums that can be reversed) and ‘periodontitis' (inflammation that results in loss of periodontal attachment)^2^^,^^25^ are increasingly prevalent. Given the irreversibility of periodontitis, and given that the impact on peoples' lives of later stage disease are more serious, further research in this field should be focused on the following key questions.

#### 5. Does telling people to remove plaque make any difference? And, if so, how can we do it better?

A great deal of time, and effort, is invested by dental professionals in chairside oral hygiene instruction and yet there is no high-certainty evidence that it is effective in preventing periodontal diseases. This situation has existed for decades. We need to explore ‘does telling people to remove plaque make any difference?'; and, if we do it, ‘how can we do it more effectively?' Four issues are grouped together because they are so closely related, and the evidence for all three is currently low/very low certainty evidence (5−a, b and d), or just good practice (5c).

Although randomised controlled trials (RCTs), may be required ultimately, the first stage would be a good critical review that might reconceptualise the role of professional oral hygiene instruction within the context of a comprehensive model of disease aetiology (including the social determinants of health). For instance, if people's behaviour is constrained by their environment, why do we invest so much time in trying to change it? However, oral hygiene is mostly a personal activity; thus, potentially easier to tackle than other issues such as diet. It may be that new research questions would arise from this work as the scope of the periodontal GDG was (rightly) rooted in existing understanding. It may be that the right questions have not yet been explored.

#### 6. Can preserving oral health or treating oral disease reduce the incidence and/or severity of systemic disease?

The relationship between systemic disease and oral health is an important issue in our ageing population, particularly in relation to periodontology. It would appear to be a growing problem given the rising prevalence of non-communicable diseases such as diabetes and cardiovascular disease. To date there is low certainty evidence that poorly controlled diabetes substantially increases the risk or progression of periodontitis; and moderate certainty evidence that diabetic control improves periodontal health.^2^ However, it raises the issue of clinical versus statistical significance (minimum clinically important difference) and possible returns on investment. Furthermore, is there an intervention (brief advice) that could be used by professionals across health and social care in support of oral health?

#### 7. What is the optimal professional management of dental implants?

As more dental implants are placed, and we recognise the incidence and impact of peri-implant disease, there is a need to inform the best protocols for their professional maintenance. Maintenance protocols to prevent peri-implant disease are generally based on cohort studies of patients attending, or not attending, for maintenance. However, there is considerable risk of confounding in these studies, as patients who do not attend for maintenance may have other habits that mitigate against good oral health. Ideally to strengthen the evidence base, large randomised controlled trials of different maintenance protocols are needed. Study arms should include ‘no maintenance' and ‘maintenance intervals' of different frequency. There is, however, an ethical complexity in conducting such research given that there are current clinical protocols (albeit based on low quality evidence) so that RCTs involving limited maintenance may be regarded as withholding ‘best practice' care; hence, it never may be feasible to do so.

### Oral cancer/head and neck cancer

Oral cancer is the most life threatening of the oral and dental conditions.^2^ The top three priority areas to be addressed relate to building evidence on primary and secondary prevention, as outlined below. Given the increasing emphasis of head and neck cancer and overlapping oral and oropharyngeal cancers, there would be benefits in broadening the scope of this guidance in future.

#### 8. What is the effectiveness of smoking cessation (including the role of vaping) in dental settings?

Smoking remains the most important risk factor for oral cancer, in addition to periodontal disease.^2^ Evidence on the effectiveness of smoking cessation in dental settings, including prescription of nicotine replacement therapy (NRT) is emerging.^2^^,^^26^ Vaping is a relatively new element of the tobacco cessation package, and it will be important to assess the emerging longer-term effects of vaping in relation to oral mucosal disease, oral cancers (and periodontal disease). Research on enhanced dental health advice in dental settings, currently being conducted with the support of NIHR, will begin to address this question.^27^

#### 9. What is the most appropriate prevention bundle of care both for preventing oral cancer and for patients following oral cancer treatment?

In addition to improving the effectiveness of interventions for smoking cessation in dental practice and given the synergistic risk of smoking with alcohol in magnifying oral cancer risk, further research is needed on the role of alcohol reduction/cessation interventions in dental settings.^28^ This research could include developing and testing combined (smoking and alcohol) interventions perhaps linking with risk assessment or prediction tools. This ought to include research to identify the barriers and facilitators to implementation of these prevention interventions.

Moreover, further research is also required to improve guidance for preventive dental care in people who have had oral cancer and where their cancer treatment (including surgery, and/or chemotherapy, and/or radiotherapy) puts them at a higher risk of oral and dental diseases. Tailored prevention interventions could be developed and tested among this high-risk group.

#### 10. How can referral pathways for patients with suspected oral cancer be improved?

For the first time DBOHv4 considered early identification and diagnosis of cancer and pathways to care.^2^ Diagnosis and appropriate management require patients to attend specialist hospital services. However, people diagnosed with oral cancer are more likely to be irregular dental attenders and this may contribute to advanced stage diagnosis. Improving referral pathways of patients with suspected oral cancer who do not routinely visit a dentist remains a challenge. There is a head and neck cancer healthcare systems analysis currently being undertaken in the international multicentre HEAD and neck cancer in South America and Europe (HEADSpAcE) study,^29^ which may provide further insights on this issue.

### Tooth wear

The term ‘tooth wear' has been embraced to cover all aspects of attrition, abrasion, and erosion by mechanical and chemical factors, given their inter-related nature.^2^ It is a natural feature of ageing and more evident now than in the past as most adults retain at least some of their natural dentition into older age. However, it is of greatest concern when it is accelerated or non-age related.^2^ The top three priority areas to be addressed are as follows.

#### 11. What are the dietary causes of dental erosion?

Population-based longitudinal epidemiological studies including dietary analysis and perhaps salivary flow analysis, would be helpful to develop an understanding of the ‘normal' rate of wear over time. This would assist with a range of issues including the identification of a hierarchy of foods/drinks associated with the likelihood of tooth wear, and the role of protective food/drinks such as milk/yoghurt/cheese/chewing gum. It would be helpful to include analysis of relative harms and wider health benefits of various types of food/drinks, e.g., fruit/milk, etc. The impact of frequency of fruit consumption and fruit juice on the rate of development of enamel erosion, especially in children and young people is important to understand to assist with balancing health benefits and risks.^30^ Longitudinal research is also needed to help assess rate of tooth wear, its impact on quality of life, and the relative cost and benefits of associated dental care.

An in-depth analysis of risk factors for individuals for whom fruit is perceived as the only identified risk factor would be beneficial given that such people are more likely to be preventively orientated in general and perhaps end up in specialist settings. Much of the research to date has been conducted in specialised secondary care clinical settings where the patient base may not be representative of the general population. Consideration should therefore be given to the settings and patient types in the above research on these issues.

#### 12. What preventive interventions work for tooth wear in vivo?

Evaluating *in vivo* interventions to prevent tooth wear requires RCTs conducted in community settings or with primary care patients rather than with people referred to secondary care centres for tooth wear management. Clinical studies (not *in vitro* or *in situ)* should include preventive management using a range of toothpastes containing sodium fluoride and/or topical fluoride in mouth rinse or varnish containing various fluoride formulations. It is important that toothpastes seeking to prevent tooth wear contribute to prevention of dental caries as part of their wider oral health care.

#### 13. What index or indices are most appropriate for screening for and monitoring of tooth wear in a primary care setting?

This will require validation in a primary dental care setting to assess whether one tool (screening and monitoring) has superiority over others when applied in this context. The type of index used has a significant impact on the prevalence of tooth wear recorded. This should include any training necessary and the time to complete the examination in a dental practice setting as part of the clinical assessment.

### Behaviour change

Important evidence on supporting behaviour change is emerging across health and healthcare and it received greater emphasis in DBOHv4.^2^ It is a complex field; and, therefore, vital to recognise that merely telling people, including patients, to change their behaviour is insufficient to achieve change, given the array of factors that influence behaviour. Much of oral healthcare involves regular personal self-care, or care of a vulnerable dependent. Furthermore, dental team members require support to change clinical care in light of clinical guidance. The three priority areas to be addressed are as follows:

#### 14. What brief behaviour change interventions (for delivery by primary dental teams) can reduce consumption of sugar in different groups?

While specific behaviour-change interventions have been developed for some oral health-related behaviours there has been little progress on tackling sugar consumption. Further research in this field is required as outlined above in Question 4. This research should begin with gaining an understanding of the determinants of sugar consumption of specific sections of the UK population, including young children, teenagers, and older people, then progress to develop a brief intervention based on behaviour change theory. This intervention needs to be acceptable (to patients and dental teams) for use in primary dental care and evaluated appropriately. It is unlikely that a traditional RCT alone will be sufficient to investigate important aspects such as implementation within dental settings, acceptability to patients, and the mechanisms of action of such an intervention.

#### 15. How best to optimise the implementation of behaviour change interventions using primary dental teams using skill mix

As alluded to above, there are also gaps in understanding how existing and any new behaviour change interventions can be best implemented, particularly within primary care and using the core and extended skills of the whole dental team. Such future research must draw on the principles of implementation science to examine and address the key factors that influence implementation at different levels (external, internal, and individual).^31^

#### 16. What intervention(s) can improve routine attendance by vulnerable (inclusion groups) within the population

A key oral health behaviour is dental attendance. Dental attendance offers the opportunity for early diagnosis and detection of disease and provision of preventive interventions, including advice. Patient's dental visiting behaviours are complex and likely to be influenced by dental service factors.^32^^,^^33^ Many vulnerable groups in society, with overlapping risk factors for poor health, face additional challenges in accessing healthcare.^34^ The term ‘inclusion groups' is increasingly being used in recognition that people who are socially excluded, typically experience multiple overlapping risk factors for poor health, such as poverty, violence and complex trauma.^34^ However, building on evidence,^32^^,^^35^ a theory-based behaviour change intervention promoting routine dental care positioned at the ‘teachable moment' of urgent care use shows promise.

#### 17. Other strategic issues

Reflecting on population needs, there is a paucity of evidence on preventive care for vulnerable adults, and how they can best be supported holistically, integrating oral disease prevention with general health. This is particularly important among older adults which represent the fastest growing section of the population.^36^

It was noted that there has been little or no contribution of risk factor management by oral healthcare to general health, i.e., oral health to general health, as well as general health to oral health.

In general, there is a need to build higher quality oral and dental evidence to provide strong recommendations; and, also, to build evidence on implementation of preventive guidance, at system, practice, and patient level, including the use of dental team skill mix to deliver prevention in line with their scope of practice.

## Discussion

This article focuses on specific areas in relation to the principal diseases and conditions of the oral cavity, and behaviour change, because the guidance in DBOH is for dental teams. It highlights an increasing need to develop evidence across the life course and notes the lack of evidence of implementation within health systems. Much of this requires a paradigm shift in healthcare.

The James Lind Alliance top ten areas for oral health research^9^ covered many of the same topics as this paper, but with a focus at community and population levels rather than the individual. Hence, this list complements the James Lind Alliance list with some overlap including the ‘delivery of oral hygiene for people with additional care needs', consideration of the ‘most effective ways of increasing early detection/diagnosis of oral cancer' and the importance of effective ‘communication'.^9^

The contribution of risk factor management by oral healthcare to general health, i.e., oral health to general health, as well as general health to oral health, would benefit from further research. Furthermore, given that there is a policy level focus on tackling obesity and overweight, efforts could be made to include caries as an outcome in future research in this area, providing evidence on integrated action for health with potentially little additional funding. While strategic plans and future contracting arrangements may highlight the need for more preventive care,^37^^,^^38^^,^^39^ it remains a reality that unless systemic barriers to delivery of prevention on the front line of dentistry are addressed, there will be minimal change. And change to the management risk and protective factors is urgently required, given the findings of the most recent Adult Oral Health Survey for England 2023^40^ which show that untreated disease, notably dental caries, has increased.

Several limitations of this paper need to be acknowledged. Firstly, the DBOHv4 development process did not set out to look at gaps in evidence, but rather to search for the body of evidence relating to prevention in clinical settings. Secondly, the process was focused on systematic reviews; the body of primary evidence was not reviewed, except in specific instances where no secondary evidence existed. However, given the wide range of academic and clinical experts involved, in the process and the potential for stakeholders to submit additional evidence during the final engagement process (based on the penultimate version), it is unlikely that strong evidence will have been missed.

## Summary and next steps

This multistage collaborative initiative facilitated a rigorous guideline development process, reviewing and compiling contemporary evidence. The process highlighted important gaps in the research evidence which should be prioritised in support of future updates. Given the global and national burden of untreaded oral disease, much of which is preventable, it is important that policymakers and funders prioritise oral and dental research. Shaping future national research strategies to embrace oral health research is a priority to support tacking the global burden of oral disease. We encourage clinicians and researchers nationally and internationally, to find innovative ways to work with researchers and others seeking to address these priority questions in a rigorous manner and funders to prioritise oral health given its prevalence. Some of this research should preferably be undertaken in primary care settings, given that most dentistry is provided in primary care settings and be integrated with wider health research. Such research should be a priority for NIHR and other research funding bodies.
